# Supplementary material for: Exploring alternative cytokines as potential biomarkers for latent tuberculosis infection in pregnant women
Source: PLoS One. 2022 Jul 8;17(7):e0270552. doi: 10.1371/journal.pone.0270552 (PMC9269918; doi:10.1371/journal.pone.0270552)
Supplement: Attachment — Submitted filename: Response to Reviewers.docx [file pone.0270552.s002.docx]

**Response to Reviewers**

To: Wenping Gong, Ph.D.

Academic Editor

PLOS ONE

Dear Dr. Wenping Gong,

Subject: Submission of revised paper number: PONE-D-22-12130

We would like to thank you for your email on May 28^th^ 2022 enclosing the academic editor’s and reviewer’s constructive comments to improve the quality of our manuscript. We have carefully reviewed the comments and have revised the manuscript accordingly. Our responses for the comments are given in a point-by-point manner as indicated below. All changes to the manuscript are highlighted in red by using “track changes” in the Microsoft Word software.

We have also updated our cover letter by adding funding information and competing interest declaration. In addition, we have ensured that all figures were under PLOS requirement by using Preflight Analysis and Conversion Engine (PACE) digital diagnostic tool. The updated cover letter and figures have been resubmitted as an attachment file to editorial manager platform.

We hope that the revised manuscript and our accompanying responses are sufficient to prepare our manuscript suitable for publication. We look forward to hearing from you in due course.

Yours Sincerely,

Agnes Rengga Indrati

Department of Clinical Pathology

Faculty of Medicine, Universitas Padjadjaran/Hasan Sadikin General Hospital, Indonesia

agnes.indrati@unpad.ac.id

+62 811-2257-928

**A. Comments from academic editor**:

1. Please ensure that your manuscript meets PLOS ONE's style requirements, including those for file naming. The PLOS ONE style templates can be found at
   https://journals.plos.org/plosone/s/file?id=wjVg/PLOSOne_formatting_sample_main_body.pdf and https://journals.plos.org/plosone/s/file?id=ba62/PLOSOne_formatting_sample_title_authors_affiliations.pdf

**Response**: We are thankful for your input. Therefore, we have revised the file name according to the guidelines.

2. Thank you for stating the following in your Competing Interests section: "No". Please complete your Competing Interests on the online submission form to state any Competing Interests. If you have no competing interests, please state "The authors have declared that no competing interests exist.", as detailed online in our guide for authors at http://journals.plos.org/plosone/s/submit-now
   This information should be included in your cover letter; we will change the online submission form on your behalf.

**Response:** We are thankful for your support. In addition, we also revise the competing interest declaration sentence in the cover letter to "The authors have declared that no competing interests exist."

3. Please amend the manuscript submission data (via Edit Submission) to include author Agnes Rengga Indrati

**Response:** We are thankful for your comment. Therefore, we have checked and made sure that Agnes Rengga Indrati is included as one of the authors and also as corresponding author as indicated in a screenshot picture below.

4. Your ethics statement should only appear in the Methods section of your manuscript. If your ethics statement is written in any section besides the Methods, please delete it from any other section.

**Response:** We are thankful for your valuable comment. We have checked and made sure that our ethics statement is only appear in the Methods section of our manuscript. In addition, we also removed the attached letter that consists of ethical statement from the uploading section in editorial manager platform.

5. Please ensure that you refer to Figure 3 in your text as, if accepted, production will need this reference to link the reader to the figure.

**Response:** We are thankful for your valuable comment. We have revised the manuscript to refer to Figure 3 in the manuscript text.

6. Please review your reference list to ensure that it is complete and correct. If you have cited papers that have been retracted, please include the rationale for doing so in the manuscript text, or remove these references and replace them with relevant current references. Any changes to the reference list should be mentioned in the rebuttal letter that accompanies your revised manuscript. If you need to cite a retracted article, indicate the article’s retracted status in the References list and also include a citation and full reference for the retraction notice.

**Response:** We are thankful for your valuable comment. We have checked the reference list and ensured that all references are correct and not retracted.

**B. Comments from reviewers**:

1. **Reviewer #1**: The topic selection of this article has certain theoretical significance and application value. The current situation and problems of tuberculosis latent infection in pregnancy are summarized, and explored the possibility of IP-10, IL-10 and IL-2 to judge LTBI in pregnancy, with certain innovation and operability, and the research direction is clear. The article is written standard, the content is relatively complete, the statistical method is correct, the chart drawing is qualified, prominent focus, has certain clinical significance. The drawback is that the comparison between pregnant women and the general population is not made, and the sample size is also small, and the persuasion is limited.

**Response:** We are grateful for your valuable comments. We acknowledge that our study had certain limitations, including small sample size and also the difficulty to find latent tuberculosis cases in pregnant women. On the other hand, due to the difference in immunological condition between pregnant and general population, especially cytokine expression that may result in diverse variability, we only included pregnant women population in our study.

2. **Reviewer #2**: This manuscript represented an interesting piece of work about the diagnosis of LTBI in pregnant women. Some minor flaws have been found and listed below after reviewed the manuscript. I suggest to accept the manuscript in the reivised form.
   1. Why there are two age categories listed in table 2? The result of the diagnostic biomarkers for LTBI in pregnancy has nothing to do with the age categories. Furthermore, the data of the two age categories is not used for analysis in this paper. It is recommended not to group age categories here.

2. As the author said: “The gestational age data of the study subjects were not well-spread where the first semester was consist of only one subject.” Therefore, more pregnant women in trimester 1 should be involved in this study.

**Response:**

1. We are grateful for your valuable input; therefore, we will revise the manuscript by deleting group age categories.
2. We acknowledge that the gestational age data is the limitation of this study; however, the subject for this experiment were collected randomly to avoid population bias. Consequently, there was a chance that we may acquire lesser samples from trimester 1 subject.
